# Cysteamine-supplemented diet for cashmere goats: A potential strategy to inhibit rumen biohydrogenation and enhance plasma antioxidant capacity

**DOI:** 10.3389/fvets.2022.997091

**Published:** 2022-10-10

**Authors:** Tiecheng Wu, Jianyong Liang, Tao Wang, Ruoyang Zhao, Yuejun Ma, Yulin Gao, Shengguo Zhao, Guoshun Chen, Bin Liu

**Affiliations:** ^1^College of Animal Science and Technology, Gansu Agricultural University, Lanzhou, China; ^2^Inner Mongolia Academy of Agricultural and Animal Husbandry Sciences, Hohhot, China

**Keywords:** cysteamine, rumen microbiome, non-targeted metabolomic, biohydrogenation, antioxidative capacity, GH-IGF-1 axis, cashmere goat

## Abstract

Cysteamine (CS), as a feed supplement, can increase the level of growth hormone (GH) in the blood, promote animal growth. However, little attention has been paid to the effects of CS on the rumen microbiome and metabolic profile in cashmere goats. This study aimed to assess the effects of rumen microbiota, metabolites, and plasma antioxidative capacity induced by CS supplementation in cashmere goats. We selected 30 Inner Mongolia white cashmere goat ewes (aged 18 months), and randomly separate the goats into three groups (*n* = 10 per group) to experiment for 40 days. Oral 0 (control group, CON), 60 (low CS, LCS), or 120 mg/kg BW^−1^ (high CS, HCS) coated CS hydrochloride every day. Using 16S and internal transcribed spacer (ITS) rRNA gene amplicon sequencing, we identified 12 bacterial and 3 fungal genera with significant changes among the groups, respectively. We found a significant increase in rumen NH_3_-N and total volatile fatty acid (TVFA) concentrations in the LCS and HCS groups compared with the CON. With untargeted LC–MS/MS metabolomics, we screened 59 rumen differential metabolites. Among the screened metabolites, many unsaturated and saturated fatty acids increased and decreased with CS treatment, respectively. CS supplementation increased the levels of plasma total antioxidant capacity (T-AOC), glutathione peroxidase (GSH-Px), superoxide dismutase (SOD), GH, and insulin-like growth factor-1(IGF-1). Spearman correlation analysis revealed that the abundance of *U29-B03, Lactococcus*, and *Brochothrix* were positively associated with the levels of δ2-THA, TVFA and antioxidant capacity. In conclusion, CS significantly affected rumen microbiota and fermentation parameters, and ultimately inhibited the biohydrogenation of rumen metabolites, enhanced plasma antioxidant capacity, and regulated some hormones of the GH–IGF-1 axis. This study provides an overall view into the CS application as a strategy to improve health production in cashmere goats.

## Introduction

Goat (*Capra hircus*) can be divided into dairy type, cashmere type, meat type and dual-purpose type according to their economic use. Cashmere goats have the advantages of roughage resistance, strong adaptability and good cashmere quality, and are mainly distributed in arid and semiarid regions of Asia and Africa. Compared with sheep, cashmere goats also exhibit greater fibre digestion ability and adaptability (1, 2). Low production performance remains an obstacle to the development of the cashmere industry. Nutritional alteration is increasingly used in animal husbandry to increase production (3, 4). Rumen microorganisms, mainly composed of bacteria, fungi, archaea, ciliated protozoa and viruses, can break down fibre, polysaccharides and other nutrients in feed, produce volatile fatty acids, microbial proteins, and vitamins, and provide nutrients to meet the host's requirement (5). The supplementation of oregano essential oil (6), yeast (7, 8), cysteamine (9, 10), etc. to ruminant diets can alter rumen microflora and fermentation patterns, and improve rumen digestibility, thereby improving animal nutritional status and promoting animal growth.

Cysteamine (CS; β-mercaptoethylamine, HS-CH_2_-CH_2_-NH_2_) is a bioactive peptide endogenously derived from coenzyme A degradation. The free thiol group in CS can react with the disulphide bonds of peptides and proteins such as somatostatin (SS), and interferes with their function (11). The CS participates in the growth hormone–insulin-like growth factor-1 axis (GH–IGF-1 axis) (12), shifts rumen microflora and inhibits rumen methane production (13, 14). Barnett's study (15) found that oral CS hydrochloride was able to cause increases in feed efficiency, wool and body growth with no additional consumption of feed in Merino × Dorset lambs. In a subsequent study, Rui Hu et al. (8) noted that the adding CS to diet increased the ruminal epithelial volatile fatty acid absorption gene expressions and improved nutritional status of growth-retarded yaks. In another study, Dietary coated CS supplementation may have a compensatory effect on muscle nutrients deposition in finishing pigs fed a reduced trace minerals diet (16).

However, the rumen being an important nutrient digestion organ of ruminants, how feeding CS will affect its microbiota and the rumen fermentation function is still not well understood. We hypothesise that the supplementation of CS will alter rumen microflora and rumen metabolism, regulated some hormones of the GH–IGF-1 axis and improve blood antioxidant capacity in cashmere goats. Therefore, the effects of CS levels in diets on rumen microbiota, metabolites, and blood hormone and antioxidant indicators of cashmere goats were investigated in the present study, aiming to provide new insights into the underlying mechanisms of CS affecting the rumen function and a theoretical basis for the use of CS for cashmere goat use.

## Materials and methods

### Animals

This study was conducted at the Alxa White Cashmere Goat Breeding Farm, in the Inner Mongolia Autonomous Region of China from September 1, 2020, to October 10, 2020. All procedures used in this study were approved by the Animal Welfare and Ethical Committee of the Inner Mongolia Academy of Agriculture and Animal Husbandry Sciences (Inner Mongolia, China). Thirty Inner Mongolia white cashmere goat ewes, with an initial average body weight of 30.53 ± 2.05 kg (aged 18 months), were randomly assigned into three groups (*n* = 10), and there were no statistically significant differences in initial body weight among the three groups (*P* = 0.243). One group was given oral 50 ml of distilled water (placebo) serving as a control (CON), and the other two groups were separately given oral 50 ml of distilled water containing 60 mg/kg BW^−1^ coated CS hydrochloride (LCS) and oral 50 ml of distilled water containing 120 mg/kg BW^−1^ coated CS hydrochloride (HCS). During the experimental period (40 days), cashmere goats were allowed graze and drink water *ad libitum* and supplementary feeding management, with 0.25 kg/d supplementary feed (ingredients and nutrition levels are shown in Supplementary Table 1) per goat to meet the nutritional needs of the goats. The concentration and frequency of CS administration were based on previous studies (10, 14). Coated CS hydrochloride, containing 30% CS hydrochloride with starch and dextrin for stabilisation, was supplied by Qingdao Runbot Biotechnology (Qingdao, China). The composition of the primary vegetation in the area was *Artemisia frigida* Willd, *Potaninia mongolica Maxim*., *Sarcozygium xanthoxylon* Bunge, *Reaumuria songarica (Pall.)* Maxim., *Haloxylon ammodendron (C. A. Mey.)* Bunge, *Caragana Korshinskii* Kom, etc.

### Rumen fluid sampling

On day 41, ruminal fluid samples were collected through the oesophagus before morning feeding using an oral stomach tube connected to a vacuum pump. The tube was inserted into the central rumen to reduce contamination (17). Approximately 50 mL was collected from six cashmere goats randomly selected from each group. The samples were individually filtered through four layers of sterile cheesecloth to separate rumen fluids from solids. The rumen fluid was transferred to two 5 mL tubes that were immediately frozen in liquid nitrogen and stored at –80°C for DNA extraction and metabolomic analysis. Another 10 mL was stored at –20°C for the evaluation of fermentation parameters.

### Fermentation parameters

The pH of the ruminal fluid was measured immediately after filtering the sample using a portable pH meter (FE28-TRIS, Mettler Toledo, Switzerland). The concentration of ammonia nitrogen (NH_3_-N) was measured using a phenol-hypochlorite assay with visible spectrophotometry (UV-4802, Unico, China), as previously described (18). Total volatile fatty acid (TVFA) was determined using a gas chromatograph (GC-2010, Agilent Technologies) fitted with an AT-FFAP capillary column (50 m × 0.32 mm × 0.25 μm) (6).

### Bacteria and fungi sequencing and analysis

Bacterial and fungal profiling was conducted by OE Biotech (Shanghai, China) (http://www.oebiotech.com). Microbial genomic DNA was extracted from the rumen content using a QIAamp 96 PowerFecal QIAcube HT kit (Qiagen, Hilden, Germany) according to the manufacturer's instructions. All operations were performed under aseptic conditions. DNA concentration and integrity were measured using a NanoDrop 2000 spectrophotometer (Thermo Fisher Scientific, Waltham, MA, USA) and agarose gel electrophoresis, respectively. Bacterial sequencing targeted the V3–V4 regions of 16S rRNA using the universal primer pair 343F (5′-TACGGRAGGCAGCAG-3′) and 798R (5′-AGGGTATCTAATCCT-3′) (19); fungal sequencing targeted the internal transcribed spacer (ITS) region ITS1 with the universal primer pair ITS1F (5′-CTTGGTCATTTAGAGGAAGTAA-3′) and ITS2 (5′-GCTGCGTTCTTCATCGATGC-3′) (20). PCR sequencing was performed on an Illumina NovaSeq 6000 with two paired-end read cycles of 250 bp each (Illumina, San Diego, CA, USA; OE Biotech). Amplicon quality was visualised by gel electrophoresis. The PCR products were purified using Agencourt AMPure XP beads (Beckman Coulter, USA) and quantified using a Qubit dsDNA assay kit, with concentrations adjusted for sequencing. Paired-end reads were pre-processed using Trimmomatic software (21) to detect and cut off ambiguous bases (N) and sequences with an average quality score below 20. Paired-end reads were assembled using FLASH software (22) using the following parameters: 10 bp of minimal overlap, 200 bp of maximum overlap, and 20% of maximum mismatch rate. Ambiguous homologous sequences or reads below 200 bp were removed. Reads with 75% of bases above Q20 were retained using QIIME software (version 1.8.0) (23). Reads with chimaeras were detected and removed using UCHIME (version 2.4.2) (24). Clean reads were subjected to primer sequence removal and clustering to generate operational taxonomic units (OTUs) using VSEARCH software with a 97% similarity cut-off (25). Representative reads for each OTU were selected (using the QIIME package), annotated, and blasted against the Silva database (version 132) using RDP classifier (confidence threshold of 70%) (26). Alpha diversity was estimated for rumen content samples and includes the Chao1 index (27), Good's coverage, Simpson index, and Shannon index (28). The binary-Jaccard distance metric was used for principal coordinate analysis (PCoA) with QIIME software.

### Metabolomic processing and analysis

For each rumen fluid sample, 500 μL was added to a 1.5 mL Eppendorf tube and centrifuged at 13 000 rpm and 4°C for 15 min. We added 100 μL of the supernatant, 20 μL of 2-chloro-l-phenylalanine (0.3 mg/mL) dissolved in methanol as an internal standard, and 400 μL of methanol:acetonitrile (2:1, v:v) to a 1.5 mL Eppendorf tube. The mixture was vortexed for 1 min, ultrasonicated on ice for 10 min, and stored for 30 min at – 20°C. The mixture was centrifuged for 10 min at 13 000 rpm and 4°C. The supernatant (200 μL) was transferred to an LC–MS vial, dried using a freeze-concentration centrifugal dryer, and mixed with a 300 μL methanol/water mixture (1:4, v/v). The mixture was vortexed for 30 s, ultrasonicated for 3 min, stored for 2 h at – 20°C, and centrifuged at 13, 000 rpm and 4°C for 10 min. The supernatant was filtered through a 0.22 μm microfilter, transferred to an LC–MS vial, and stored at – 80°C. QC samples were prepared by mixing aliquots of all the samples.

An Acquity UHPLC system (Waters Corporation, Milford, CT, USA) coupled with an AB SCIEX Triple TOF 6600 (AB SCIEX, Framingham, MA, USA) was used to analyse the metabolic profiles. An Acquity UPLC HSS T3 column (100 × 2.1 mm, 1.8 μm) was used in both positive and negative modes. Binary gradient elution consisted of (A) water (containing 0.1% formic acid, v/v) and (B) acetonitrile (containing 0.1% formic acid, v/v), and separation was achieved using the following gradient: 0 min, 5% B; 2 min, 20% B; 4 min, 60% B; 11 min, 100% B; 13 min, 100% B; 13.5 min, 5% B and 14.5 min, 5% B; flow rate, 0.35 mL/min; and column temperature, 45°C.

The original LC–MS data were processed using Progenesis QI version 2.3 (Nonlinear Dynamics, Newcastle, UK) for baseline filtering, peak identification, integration, retention time correction, peak alignment, and normalisation. Compound identification was based on the precise mass-to-charge ratio (M/z), secondary fragments, and isotopic distribution using the Human Metabolome Database (HMDB), Lipidmaps (version 2.3), Metlin, EMDB, PMDB, and self-built databases. The extracted data were processed by removing any peaks with a missing value (ion intensity = 0) in more than 50% of groups. Compounds with scores below 36 (out of 60) were deemed inaccurate and removed. A data matrix was created from the positive and negative ion data and analysed using a principal component analysis (PCA) using R software (The R foundation, Vienna, Austria). Orthogonal partial least-squares discriminant analysis (OPLS-DA) was used to assess the variation in metabolites among the groups.

### Plasma sampling

On day 41, Blood samples were collected from the jugular vein using 5 mL heparin sodium blood collection tubes before morning feeding. The 5 mL of blood samples were collected from each cashmere goat (Rumen fluid and blood are collected from the same individual). The samples were kept on an ice bath until centrifugation at 3000 × *g* for 10 min at 4°C, and the plasma was separated and stored at – 20°C.

### Hormone assays

Plasma growth hormone (GH), insulin-like growth factor-1 (IGF-1), insulin (INS), total sulfhydryl (T-SH), and SS levels were measured using an enzyme-linked immunosorbent assay (Beijing Sinouk Institute of Biological Technology, Beijing, China) and absorbance was measured using a microplate reader (Diatek DR-200BS enzyme analyser, China), all according to the manufacturer's instructions.

### Measurement of plasma antioxidative activity and enzymes

We measured total antioxidant capacity (T-AOC) using the 2,2-azino-bis-3-ethylbenzothiazoline-6-sulfonic acid (ABTS) method, needed 10 μL of plasma (29), total superoxide dismutase (SOD) activity using the xanthine method, needed 18 μL of plasma (30), catalase (CAT) activity using the ammonium molybdate method, needed 20 μL of plasma (31), and glutathione peroxidase (GSH-Px) activity using colorimetry, needed 20 μL of plasma (31). All enzyme assays were conducted using commercial kits (Beijing Sinouk Institute of Biological Technology; DR-200BS enzyme analyser, Diatek, China).

### Biochemical marker of plasma oxidative stress

The amount of plasma aldehyde products generated by lipid peroxidation was measured as malondialdehyde (MDA) content using the thiobarbituric acid method, needed 100 μL of plasma (30) (Beijing Sinouk Institute of Biological Technology).

### Statistical analysis

Rumen fermentation parameters and plasma marker data are expressed as the mean and standard error of the mean (SEM). Comparisons were made by one-way analysis of variance (ANOVA) and Duncan's multiple-range test using SPSS (version 19.0, IBM SPSS, Chicago, IL, USA). Statistical difference was, respectively declared as significant or highly significant at *P* < 0.05 or *P* < 0.01, while trend was discussed at 0.05 <*P* ≤ 0.10.

## Results

### Rumen fermentation in response to CS supplementation

The NH_3_-N concentrations increased (*P* = 0.022) with CS dose. Compared with the CON group, TVFA was higher (*P* = 0.013) and the molar proportions of isobutyrate (*P* = 0.018), valerate (*P* = 0.027), and isovalerate (*P* = 0.012) were lower in the LCS group. The pH (*P* = 0.305), molar proportions of acetate (*P* = 0.283), propionate (*P* = 0.621), and butyrate (*P* = 0.730), and acetate:propionate ratio (*P* = 0.556) were not affected by the treatments (Table 1).

**Table 1:** Effects of CS on rumen fermentation parameters in cashmere goats.

**Items**	**Treatment** ^**1**^			**SEM**	***p*-value**
	**CON**	**LCS**	**HCS**		
pH	6.91	6.72	6.92	0.034	0.305
NH_3_-N (mg/mL)	0.40^b^	0.48^ab^	0.55^a^	0.023	0.022
TVFA^2^ (mmol/L)	102.12^b^	116.68^a^	113.59^a^	2.286	0.013
Molar proportion, mmol/L/100 mmol/L
Acetate	69.37	70.81	71.48	0.543	0.283
Propionate	17.71	17.06	16.70	0.409	0.621
Butyrate	9.36	9.06	8.62	0.362	0.730
Isobutyrate	0.72^a^	0.57^b^	0.62^b^	0.023	0.018
Valerate	1.55^a^	1.40^b^	1.45^ab^	0.024	0.027
Isovalerate	1.30^a^	1.11^b^	1.14^b^	0.030	0.012
A:P^3^	3.98	4.20	4.33	0.127	0.556

^1^CON, control group, basal diet; LCS, low CS, basal diet plus 60 mg/kg/d coated CS hydrochloride; HCS, high CS, basal diet plus 120 mg/kg/d coated CS hydrochloride. Superscript lowercase letters indicate significant differences (*P* < 0.05); n = 6 per group.

^2^Total volatile fatty acid.

^3^The ratio of acetate mol% TVFA to propionate mol% TVFA.

#### Rumen microbiome response to CS supplementation

**Figure 1:**
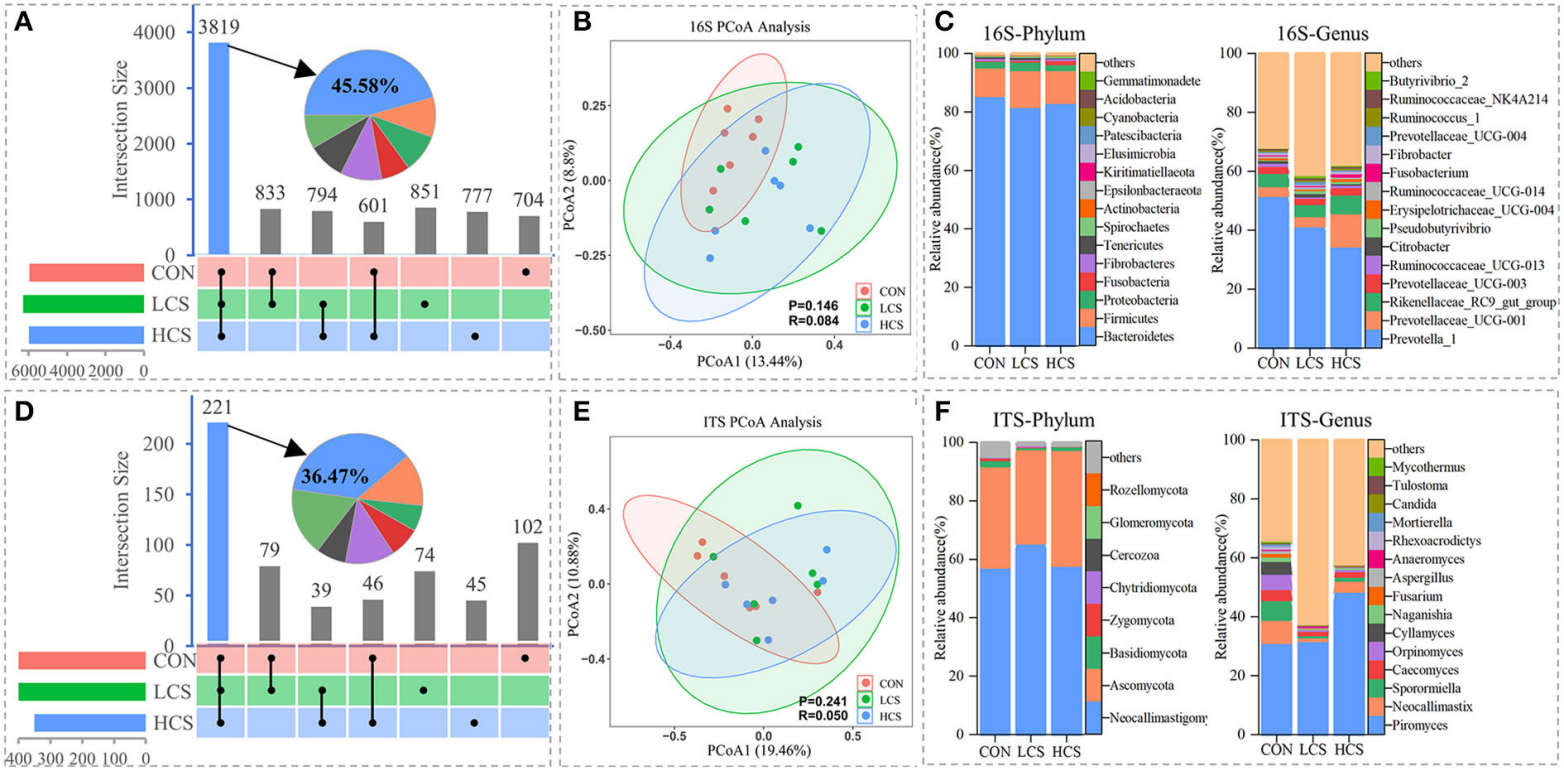
Variability in rumen microbial community composition among and within samples. **(A)** The bacterial and **(D)** fungal histograms show core operational taxonomic units (OTUs) (present in three treatments), dispensable OTUs (present in two treatments), and specific OTUs (present in one treatment). PCoA of **(B)** bacterial and **(E)** fungal communities based on the binary-Jaccard distance metric and ANOSIM. Relative abundance of **(C)** bacterial and **(F)** fungal communities at the phylum and genus level. CON, control group, basal diet; LCS, low CS, basal diet plus 60 mg/kg/d coated CS hydrochloride; HCS, high CS, basal diet plus 120 mg/kg/d coated CS hydrochloride.

For 16S rRNA bacterial and ITS rRNA fungal sequencing (*n* = 18), 1 318 291 and 806 603 clean tags were generated, respectively (the sequencing results are summarised in Supplementary Table 2). Tags with 97% similarity were grouped into 8,379 bacterial and 606 fungal OTUs. A large proportion of the microbiome was shared among groups, with 3,819 bacterial (45.58%) and 221 fungal shared OTUs (36.47%) (Figures 1A,D). The rarefaction curves almost reached a plateau (Supplementary Figure 1), The curve verifies the quality of the sequencing data. The PCoA at the OTU level showed that the three groups (CON, LCS, and HCS) were not clearly separated in bacterial (13.44 and 8.80% of the total variables, respectively; ANOSIM, binary-Jaccard metric: *P* = 0.146, R = 0.084; Figure 1B) and fungal communities (19.46 and 10.88% of the total variables, respectively; ANOSIM, binary-Jaccard metric: *P* = 0.241, R = 0.050; Figure 1E). The microbial communities based on the binary-Jaccard distance metric were similar between the LCS and HCS groups, while those of the CON group varied somewhat from those of the CS treatment groups at the OTUs level. CS supplementation did not influence bacterial and fungal alpha diversity indices, such as Chao1, Good's Coverage, Shannon, and Simpson (Supplementary Table 3).

Further analysis of microbial community structure indicated that the 16S rRNA gene sequences were affiliated with 25 phyla, 50 classes, 119 orders, 203 families, and 456 genera. The ITS rRNA gene sequences were affiliated with 8 phyla, 23 classes, 58 orders, 88 families, and 128 genera. *Bacteroidetes, Firmicutes*, and *Proteobacteria* were the three dominant bacterial phyla across all groups, accounting for 96.3–97.4% of the relative abundances of all classified bacterial sequences. The relative abundance of *Nitrospirae* was significantly higher in the LCS group than CON and HCS groups (*P* = 0.035). At the genus level, *Prevotella_1, Prevotellaceae_UCG-001, Rikenellaceae_RC9_gut_group*, and *Prevotellaceae_UCG-003* were the dominant genera (Supplementary Table 4). The relative abundance of *Prevotella_1* gradually decreased with increasing CS supplementation, whereas that of *Prevotellaceae_UCG-001* increased (Figure 1C). *Neocallimastigomycota* and *Ascomycota* were the dominant fungal phyla, together accounting for ~91.4% of the relative abundance of the total fungal sequences. At the genus level, *Piromyces*, followed by *Neocallimastix, Sporormiella, Caecomyces*, and *Orpinomyces* were the most dominant fungal genera (Figure 1F).

**Figure 2:**
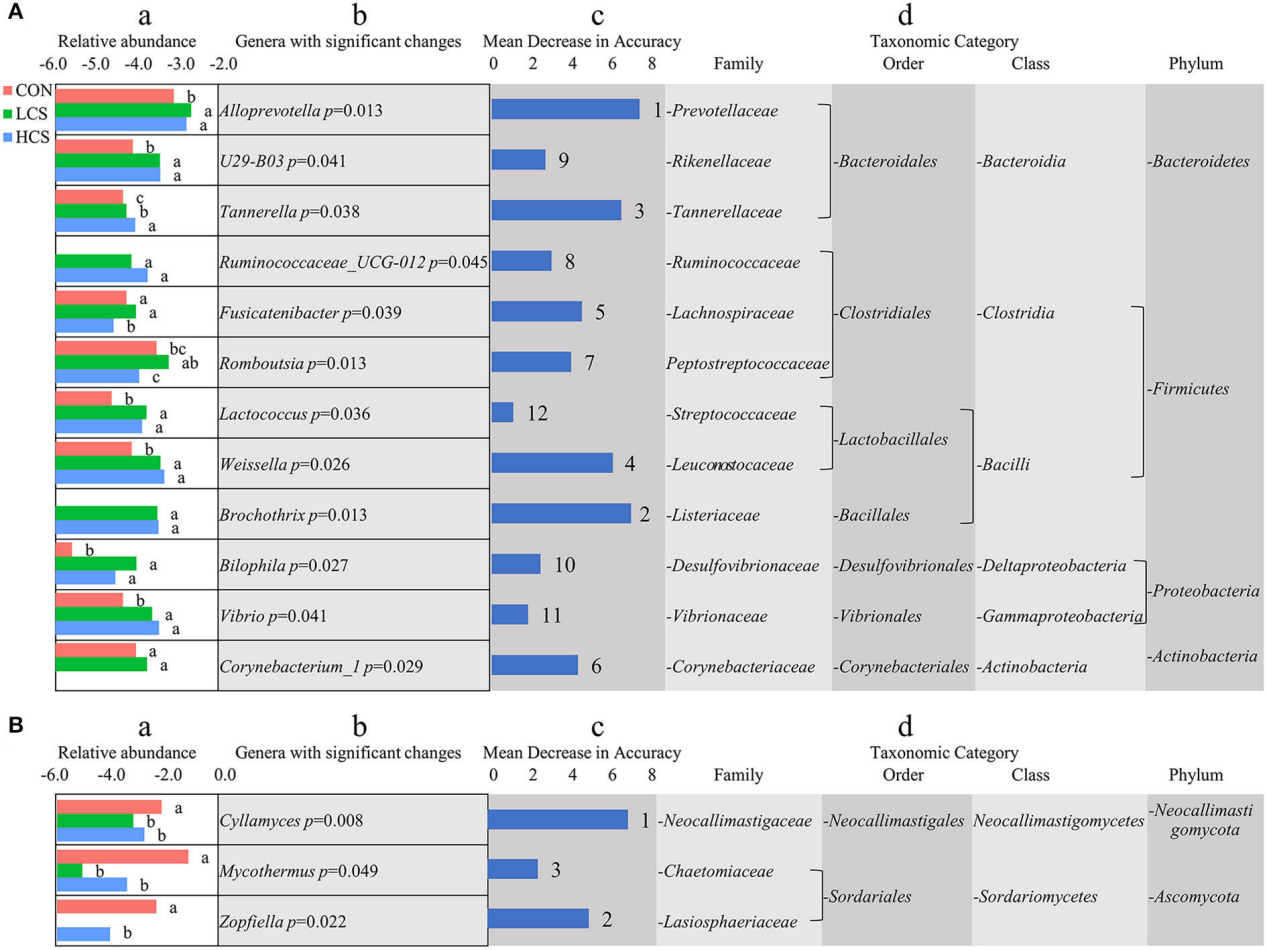
Variation in the abundance of microbial genera among groups. **(A)** Bacterial genera. **(B)** Fungal genera. (a) The log-normalised abundances for each group. Lowercase letters indicate significant differences in relative abundance. (b) Genera description and corresponding *p*-value. (c) Importance of a corresponding feature based on mean decrease accuracy (ranks are indicated to the right). (d) Taxa information for the corresponding genus. Main microbiota account for >0.01% of the total sequences in at least one of the samples. The abundance of each genus is expressed as a percentage. CON, control group, basal diet; LCS, low CS, basal diet plus 60 mg/kg/d coated CS hydrochloride; HCS, high CS, basal diet plus 120 mg/kg/d coated CS hydrochloride.

A random forest classifier method was used to evaluate the importance of genera that vary significantly in their abundance among groups. For bacteria, the abundances of 12 genera varied significantly among groups. Most of these belonged to the phyla *Bacteroidetes, Firmicutes, Proteobacteria*, and *Acidobacteria*. *Alloprevotella* (*P* = 0.013), *Brochothrix* (*P* = 0.013), *Tannerella* (*P* = 0.038), *Weissella* (*P* = 0.026), *Ruminococcaceae_UCG.012* (*P* = 0.045), *U29-B03* (*P* = 0.041), *Bilophila* (*P* = 0.027), *Vibrio* (*P* = 0.041), and *Lactococcus* (*P* = 0.036) were more abundant in the CS treatment groups and increased with CS dose. When sorted by the importance of a corresponding feature based on mean decrease accuracy, the top five genera were *Alloprevotella, Brochothrix, Tannerella, Weissella*, and *Fusicatenibacter* (Figure 2A). For fungi, the abundances of three genera varied significantly among groups and belonged to the phyla *Neocallimastigomycota* and *Ascomycota*. Compared with the CON, the relative abundances of *Cyllamyces* (*P* = 0.008), *Mycothermus* (*P* = 0.049), and *Zopfiella* (*P* = 0.022) were significantly lower in the CS treatment groups (Figure 2B).

#### Rumen metabolomic profiles in response to CS supplementation

**Figure 3:**
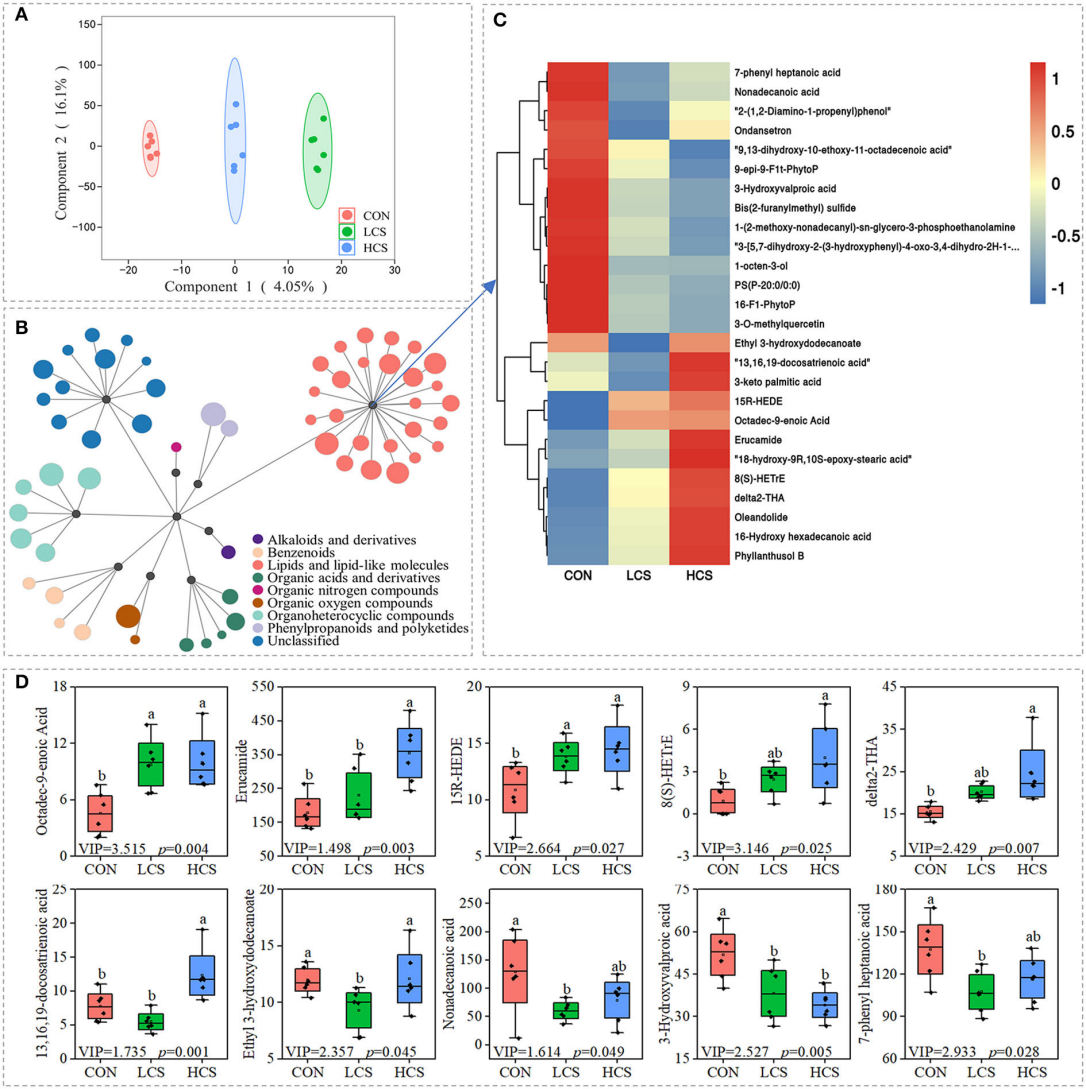
The CS-supplemented diet changed the metabolite levels in cashmere goat rumens. **(A)** OPLS-DA analysis. **(B)** Differential metabolites were clustered by super class. The size of a circle is proportional to Variable important in projection (VIP). **(C)** Thermal map clustering of metabolites presenting the relative contents of lipids and lipid-like molecules. Red and blue indicate higher and lower relative content. **(D)** Expression of fatty acids. CON, control group, basal diet; LCS, low CS, basal diet plus 60 mg/kg/d coated CS hydrochloride; HCS, high CS, basal diet plus 120 mg/kg/d coated CS hydrochloride.

To further analyse the influence of CS on microbial activity, we performed a non-targeted metabolomic analysis of rumen metabolites using liquid chromatography coupled with tandem mass spectrometry (LC–MS). A total of 4,295 metabolites were identified (Supplementary Table 5). The three groups shared the same metabolite categories, including 1,635 lipids and lipid-like molecules (the super class level is the same as below), 946 unclassified metabolites, 364 organoheterocyclic compounds, 344 organic acids and derivatives, 343 phenylpropanoids and polyketides, 271 organic oxygen compounds, 232 benzenoids, and 160 unknown compounds (Supplementary Figure 2A). Supplementary Figure 2C provides an overview of the LC–MS spectral data, including 18 rumen and QC samples, according to a PCA score plot. According to the OPLS-DA (Figure 3A), metabolic profiles varied among groups. Response permutation test plots, presented in Supplementary Figure 2B, represent the OPLS-DA model assessment parameters among the groups. The OPLS-DA models were characterised by R^2^Y = 0.997 and Q^2^ = – 0.081, indicating a good fit.

CS supplementation had a significant influence on the levels of the 59 metabolites (Variable important in projection, VIP > 1.0, *P* < 0.05) (Figure 3B and Supplementary Table 6). Many changes were observed in the levels of lipids and lipid-like molecules (fatty acids, glycerophospholipids, eicosanoids, octadecanoids, flavones, steroids, steroid derivatives, and other lipid products; Figures 3B,C). Many unsaturated fatty acids increased in the CS treatment group, together with a decrease in many saturated fatty acids. For example, the contents of octadec-9-enoic acid (*P* = 0.004), erucamide (*P* = 0.003), 15R-HEDE (*P* = 0.027), 8(S)-HETrE (*P* = 0.025), δ2-THA (*P* = 0.007), and 13,16,19-docosatrienoic acid (*P* = 0.001) increased but the contents of ethyl-3-hydroxydodecanoate (*P* = 0.045), nonadecanoic acid (*P* = 0.049), 3-hydroxyvalproic acid (*P* = 0.005), and 7-phenyl-heptanoic acid (*P* = 0.028) decreased (Figure 3D).

#### Plasma hormone responses to CS supplementation

The CS supplementation increased the concentrations of GH (*P* = 0.008), IGF-1 (*P* = 0.000), and T-SH (*P* = 0.000), decreased the concentrations of SS (*P* = 0.000), and had no effect on INS (*P* = 0.445, Table 2).

**Table 2:** Effects of CS on blood hormones in cashmere goats.

**Items^2^**	**Treatment** ^**1**^			**SEM**	***p*-value**
	**CON**	**LCS**	**HCS**		
GH (ng/ml)	4.06^b^	4.78^a^	5.23^a^	0.17	0.008
IGF-1 (ng/ml)	260.02^c^	286.03^b^	341.79^a^	8.52	0.000
INS (uIU/ml)	11.65	11.61	12.15	0.19	0.445
T-SH (umol/L)	575.81^b^	622.35^b^	768.68^a^	23.01	0.000
SS (pg/ml)	18.26^a^	15.25^b^	12.78^c^	0.61	0.000

^1^CON, control group, basal diet; LCS, low CS, basal diet plus 60 mg/kg/d coated CS hydrochloride; HCS, high CS, basal diet plus 120 mg/kg/d coated CS hydrochloride. Different superscript lowercase letters indicate significant differences (*P* < 0.05); *n* = 6 per group.

^2^GH, growth hormone; IGF-1, insulin-like growth factor-1; INS, insulin; T-SH, total sulfhydryl; SS, somatostatin.

#### Plasma antioxidant capacity in response to CS supplementation

The CS supplementation increased the concentrations of T-AOC (*P* = 0.000), GSH-Px (*P* = 0.000), SOD (*P* = 0.000), and CAT (*P* = 0.000). With the increase of CS dose, the concentration of MDA has a decreasing trend (*P* = 0.064, Table 3).

**Table 3:** Effects of CS on blood antioxidant capacity in cashmere goats.

**Items^2^**	**Treatment** ^**1**^			**SEM**	***p*-value**
	**CON**	**LCS**	**HCS**		
T-AOC (U/ml)	9.98^c^	10.92^b^	12.32^a^	0.28	0.000
GSH-Px (U/ml)	289.41^c^	309.50^b^	366.31^a^	8.04	0.000
SOD (U/ml)	80.47^c^	86.74^b^	91.18^a^	1.20	0.000
CAT (U/ml)	11.00^c^	12.25^b^	13.61^a^	0.31	0.000
MDA (nmol/ml)	5.36^a^	4.70^ab^	4.20^b^	0.21	0.064

^1^CON, control group, basal diet; LCS, low CS, basal diet plus 60 mg/kg/d coated CS hydrochloride; HCS, high CS, basal diet plus 120 mg/kg/d coated CS hydrochloride. Superscript lowercase letters indicate significant differences (*p* < 0.05); *n* = 6 per group.

^2^T-AOC, total antioxidant capacity; GSH-Px, glutathione peroxidase; SOD, superoxide dismutase; CAT, catalase; MDA, malondialdehyde.

#### Correlations of rumen microbiota, metabolomic profiles, fermentation parameters, and plasma markers

**Figure 4:**
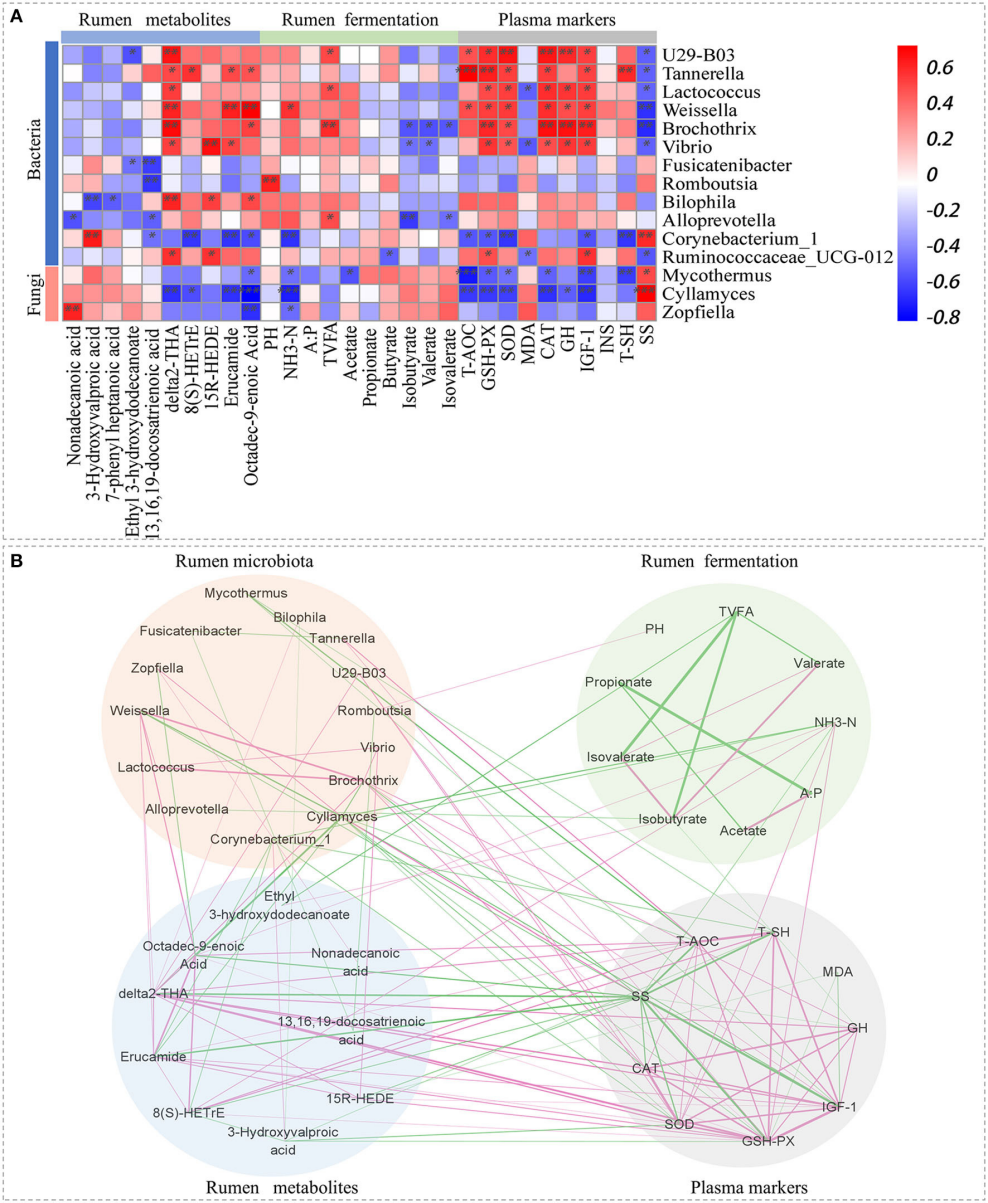
Relationship between rumen microbiota, metabolites, fermentation parameters, and plasma markers. **(A)** Spearman's rank correlation coefficients between rumen microbiota, metabolites, fermentation parameters, and plasma markers according to CS dose. Each row represents a microbial genus, each column represents a metabolite. Red represents a positive correlation, and blue represents a negative correlation; colour intensity is proportional to Spearman's rank correlation values, *P < 0.05, **P < 0.01, ***P < 0.001. **(B)** Spearman's correlation network for rumen microbiota, metabolites, fermentation parameters, and plasma markers. Only strong correlations (|r| > 0.6, *P* < 0.05) are shown.

Spearman's rank correlations among different rumen microbiota, metabolites, fermentation parameters, and plasma markers (Figure 4A) revealed that the abundance of *Tannerella, Weissella, Bilophila*, and *Ruminococcaceae_UCG-012* were positively associated with the levels of δ2-THA, 15R-HEDE, erucamide, and octadec-9-enoic acid; *Cyllamyces* and *Corynebacterium_1* were negatively associated with δ2-THA, 8(S)-HETrE, erucamide, and octadec-9-enoic acid; *Alloprevotella, U29-B03, Lactococcus*, and *Brochothrix* were positively associated with TVFA; *U29-B03, Tannerella, Lactococcus, Weissella, Brochothrix*, and *Vibrio* were positively associated with T-AOC, GSH-Px, SOD, CAT, GH, and IGF-1 but negatively correlated with SS; and *Corynebacterium_1, Cyllamyces*, and *Mycothermus* were negatively associated with T-AOC, GSH-Px, SOD, CAT, and IGF-1 but positively correlated with SS.

Spearman's rank correlation network (Figure 4B) showed a close and complex relationship among rumen microbiota, metabolites, fermentation parameters, and plasma markers. The interaction between rumen metabolites and plasma markers was particularly complicated. Finally, GSH-Px had the largest number of connexions with rumen microbiota, metabolites, and fermentation parameters (10 connexions in total), followed by SS (9 connexions in total), SOD (7 connexions in total), and IGF-1 (6 connexions in total).

## Discussion

### Rumen fermentation in response CS supplementation

We measured fermentation parameters as a proxy for microbial metabolic activity in the rumen. The pH, NH_3_-N, and VFA are important indicators of rumen fermentation and microbial health. The addition of CS to the cashmere goat diet had no significant effect on the pH of the rumen environment, this is consistent with studying on diet supplemented with CS in dairy buffalo (10). The NH_3_-N is the only direct source of microbial nitrogen in the rumen and its concentration reflects the rate at which microorganisms decompose and utilise nitrogenous substances. We found that NH_3_-N concentration increased with CS dose. It is likely that the increase in NH_3_-N concentration was due to enhanced deamination of proteins, peptides, and amino acids. In animals, CS enhances the chemical digestion of dietary nutrients by depleting SS and increasing the secretion and activity of digestive enzymes (32). The concentration of TVFA increased in the CS treatment groups, CS promoted fermentation by rumen microorganisms and increased fatty acid content. The concentrations of acetic, propionic, and butyric acids were not affected by CS, this is consistent with previous reports (10, 14).

### Rumen microbiome response to CS supplementation

The diversity of gut microbiota is closely related to animal health, metabolic capacity, and stability (33, 34). Bacterial and fungal alpha diversity indices (Chao1, Good's coverage, Simpson, and Shannon) did not vary among the—treatment groups, this is consistent with the results for buffaloes (9). Similar to previous studies (10, 35), *Bacteroidetes* and *Firmicutes* were the dominant bacterial phyla, and *Neocallimastigomycota* and *Ascomycota* were the dominant fungal phyla—both being important for the degradation and fermentation of diet biopolymers. At the genus level, we found that *Prevotella_1* was the dominant bacteria, consistent with previous reports for cashmere goats (36) and Holstein cattle (37, 38). *Prevotella_1* plays an important role in the degradation and utilisation of proteins, peptides, starch, hemicellulose, and pectin (39). Regarding fungi, *Piromyces* was the dominant genus, consistent with the findings for Saanen goats (40).

The relative abundance of *Weissella* and *Lactococcus* increased significantly with the CS treatment. These bacteria show antioxidant activity *in vitro* (41, 42) and *in vivo* (43), and are associated with lactic acid bacteria with probiotic potential (44). The relative abundance of *Weissella* and *Lactococcus* was higher in the CS treatment groups, which may be related to the antioxidant properties of CS. *Alloprevotella, Tannerella*, and *U29-B03* are Bacteroidetes genera responsible for protein hydrolysis, carbohydrate degradation, and the fermentation of amino acids to acetate (45). We found that their relative abundance increased with CS dose, similar to the findings for growth-retarded yaks (8). CS supplementation not only provides more nutrients, but also improves the efficiency of nutrient absorption and capacity for fermentation based on our findings.

### Rumen metabolomic profiles in response to CS supplementation

Rumen metabolomics approaches hold great potential for a more comprehensive understanding of the effects of functional feeds on the rumen. Hydrogen is produced by fermentation of carbohydrates, and its accumulation inhibits microorganism activity and reduces fibre degradation rates. Therefore, hydrogen transfer is essential to maintain normal fermentation (46). Biohydrogenation involves the reduction of unsaturated substances using hydrogen to form saturated metabolites (47). We observed that the CS supplementation resulted in significant changes rumen lipids and lipid-like molecules in cashmere goats, CS supplementation increased unsaturated fatty acid content, but decreased saturated fatty acid content. This was likely due to CS consuming the hydrogen produced during rumen fermentation, thus protecting against the excessive hydrogenation of unsaturated fatty acids. In ruminants, unsaturated fatty acids are converted to saturated fatty acids by the rumen microorganisms through biohydrogenation, and alleviating the hydrogenation of rumen microorganisms can allow more unsaturated fatty acids to be deposited in animal products (46, 48, 49). However, the removal or inhibition of microorganisms involved in ruminal biohydrogenation will inevitably affect the diversity and fermentative function of rumen microorganisms (50). Our findings suggest that the addition of CS to the diet of cashmere goats can mitigate the biohydrogenation of unsaturated fats in rumen without inhibiting the alpha diversity and fermentation function of rumen microbes.

### Plasma hormone responses to CS supplementation

Animal growth and development are affected by age, genotype, neuroendocrine function, and metabolism, among others (51, 52). Animal growth is largely regulated by the GH–IGF-1 axis. SS is a functional regulatory polypeptide secreted by the hypothalamus that can inhibit GHRH and GH production (53). The growth-promoting effect of CS is mainly realised by the depletion of SS (11). CS has active groups, such as sulfhydryl and amino groups, separated by two carbon atoms that can directly interact with SS molecules, change SS molecular configuration (disulphide bond), and inhibit SS biological activity. This promotes the secretion of GH from the anterior pituitary, ultimately promoting growth (53, 54). To explore the possible beneficial effect of CS on endocrine system of cashmere goats, some hormones related to the GH-IGF-1 axis, such as GH, IGF-1, INS, T-SH, SS were determined. We found that the plasma levels of GH, IGF-1, and T-SH were significantly increased, while the levels of SS were significantly decreased in the CS treatment groups. This is consistent with the results observed for red tilapia (55), lambs (56), pigs (57), sheep (58), etc. with CS supplementation, this result may be related to the lack of species specificity of SS.

### Plasma antioxidant capacity in response to CS supplementation

Aerobic metabolism generates a large amount of reactive oxygen species (ROS), ROS can lead to an imbalance in intracellular redox potential (oxidative stress) and cell damage, such as oxidative damage to DNA, lipids, and proteins (59). To explore the potential mechanism of CS on antioxidant capacity of cashmere goats, the activities of antioxidant enzymes (SOD, CAT, GSH-Px), biochemical marker of oxidative stress (MDA) and T-AOC in plasma were measured. The CS supplementation increased the concentrations of T-AOC, GSH-Px, SOD, and CAT, yet decreased the concentration of MDA, indicating that CS can improve antioxidant capacity in cashmere goats. The GSH-Px, SOD, and CAT are the primary antioxidant enzymes, and convert peroxides into less toxic or harmless substances (60). The MDA levels are commonly used as a proxy for lipid peroxidation and cellular damage *in vivo* (61, 62).The T-AOC is related to various antioxidants that protect cells from ROS and is used to evaluate the antioxidant capacity of biologically active substances. A previous study in pigs showed that CS supplementation in finishing diets reduced oxidative stress (increased GSH-Px activity and decreased MDA content) in the jejunal mucosa, CS supplementation is beneficial for intestinal health (32). The CS treatment also reduced ROS levels, MDA concentration, yet increased the GSH-Px activity in the rat brain cortex 48 h after subarachnoid haemorrhage (63). The CS can act as an antioxidant through sulfhydryl groups that can scavenge free radicals (64). The CS can break the disulphide bond in cystine to generate cysteine (a substrate for GSH synthesis), thus increasing GSH levels (11). The CS decreases lipoperoxidation and increases the carbonyl content of proteins and CAT activity (65). In addition, taurine, which can be synthesised by CS (11) could induce an increase in the activities of CAT and the enzymes involved in glutathione metabolism (66). Therefore, the increase in antioxidant capacity could also be related to an increase in taurine levels.

### Correlations of rumen microbiota, metabolomic profiles, fermentation parameters, and plasma markers

Ruminants possess a complex rumen microbial community that enables the host to digest their plant feed through microbial-mediated fermentation (5). Diet directly affects gut microbiota, ultimately affecting the gut-brain axis—a bidirectional communication system between the gut and brain mediated by hormone, immune, and neural signals (67). The microbiota produces and secretes hormones, responds to host hormones and regulates expression levels of host hormones (68). Our results showed that the abundance of *Lactococcus* was positively associated with the levels of T-AOC, GSH-Px, SOD, and CAT. This finding is consistent with that of other investigators who have observed antioxidant potential of *Lactococcus* in mice (42, 43). Several typical rumen metabolites and plasma markers were highly correlated with specific rumen microbiota, demonstrating a functional correlation among the rumen microbiome, metabolites and blood antioxidant indices. This suggests that CS can affect the rumen microflora, rumen metabolic profile, and host plasma antioxidant capacity.

## Conclusions

**Figure 5:**
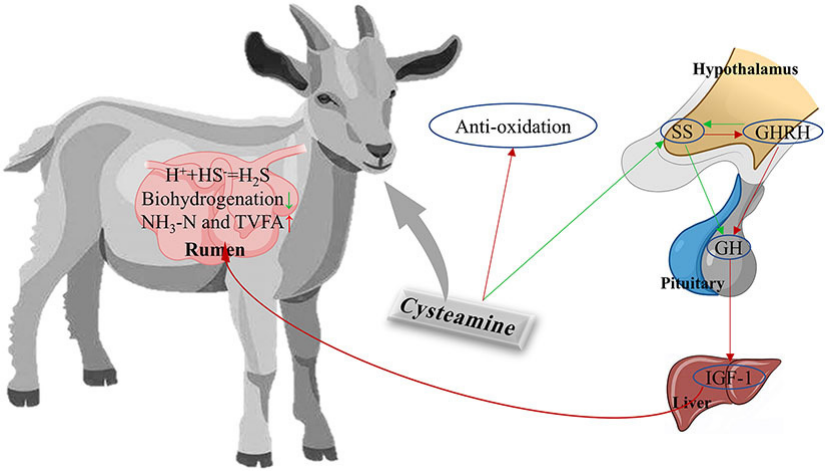
A holistic view of diet-rumen-blood interactions in response to CS supplementation in cashmere goats. Red and green arrows represent upregulated and downregulated, respectively. SS, somatostatin; GHRH, growth hormone releasing hormone; GH, growth hormone; IGF-1, insulin-like growth factor-1; SS, somatostatin.

The addition of CS to the diet of cashmere goats altered rumen microbiota, inhibited biohydrogenation, enhanced plasma antioxidant capacity, and regulated the levels of GH–IGF-1 axis-related hormones. Overall, CS exerted a positive effect on rumen function and metabolism in cashmere goats (Figure 5). We propose that CS could be a useful dietary supplement for cashmere goats grazing in desert grasslands. Significant differences in bacterial and fungal community structure, rumen metabolites, and plasma markers were observed among treatment groups (CON, LCS, and HCS). The dominant genera in the CS treatment groups could be associated with specific functions in rumen metabolism and plasma antioxidant capacity, but the exact pathway of microbiota-hormone signalling has not been elucidated. However, further studies are required to elaborate on the bidirectional communication system between rumen microbiota and endocrine hormones, and to provide a more scientific and theoretical basis for the addition of CS to the diet of cashmere goats.

## Data availability statement

The 16S rRNA and ITS rRNA gene sequences were provided and available at NCBI SRA repository with Accession Code PRJNA856420 and PRJNA856624.

## Ethics statement

The animal study was reviewed and approved by Animal Welfare and Ethical Committee of the Inner Mongolia Academy of Agriculture and Animal Husbandry Sciences, Inner Mongolia, China. Written informed consent was obtained from the owners for the participation of their animals in this study.

## Author contributions

TW, GC, and BL conceived and designed the experiments. TW, JL, TW, RZ, YM, and YG conducted the experiments and performed the statistical analysis of the experimental data. Finally, the paper was written by TW and was modified by BL and JL. All authors contributed to the article and approved the submitted version.

## Funding

This work was supported by the Inner Mongolia Agriculture and Animal Husbandry Youth Innovation Fund Project (2021QNJJM02), the Inner Mongolia Agriculture and Animal Husbandry Innovation Fund Project (2020CXJJM01), the Inner Mongolia Autonomous Region Science and Technology Project (2020GG0095), the Inner Mongolia Autonomous Region Scientific and Technological Achievements Transformation Guidance Project (2020CG0100), and Major Special Project for Cashmere Goats in Inner Mongolia Autonomous Region (2017).

## Conflict of interest

The authors declare that the research was conducted in the absence of any commercial or financial relationships that could be construed as a potential conflict of interest.

## Publisher's note

All claims expressed in this article are solely those of the authors and do not necessarily represent those of their affiliated organizations, or those of the publisher, the editors and the reviewers. Any product that may be evaluated in this article, or claim that may be made by its manufacturer, is not guaranteed or endorsed by the publisher.
