# Supplementary material for: Cysteamine-supplemented diet for cashmere goats: A potential strategy to inhibit rumen biohydrogenation and enhance plasma antioxidant capacity
Source: Front Vet Sci. 2022 Oct 10;9:997091. doi: 10.3389/fvets.2022.997091 (PMC9590691; doi:10.3389/fvets.2022.997091)
Supplement: Supplementary file 1 [file Data_Sheet_1.docx]

**Cysteamine-supplemented diet for cashmere goats: a potential strategy to inhibit rumen biohydrogenation and enhance plasma antioxidant capacity**

Supplementary materials include 2 figures and 5 tables:

Supplementary Figure 1. Rarefaction curves of the rumen microbial communities.

Supplementary Figure 2. Rumen metabolites of cashmere goats (A) Numbers of super class of identified metabolites. (B) OPLS-DA permutation test plot. (C)Total PCA plot of the rumen metabolites.

Supplementary Table 1. Ingredient and nutrient levels of the diets fed to cashmere goats (dry matter basis).

Supplementary Table 2. Summary table of tags (bacteria and fungi) distribution in the sample.

Supplementary Table 3. Alpha diversity of the rumen microbiota in cashmere goats.

Supplementary Table 4. The relative abundances of all classified bacterial and fungal sequences.

Supplementary Table 5. 4295 Metabolites information overview table.

Supplementary Table 6. 59 differential metabolites obtained among groups (CON, LCS, and HCS).

Supplementary
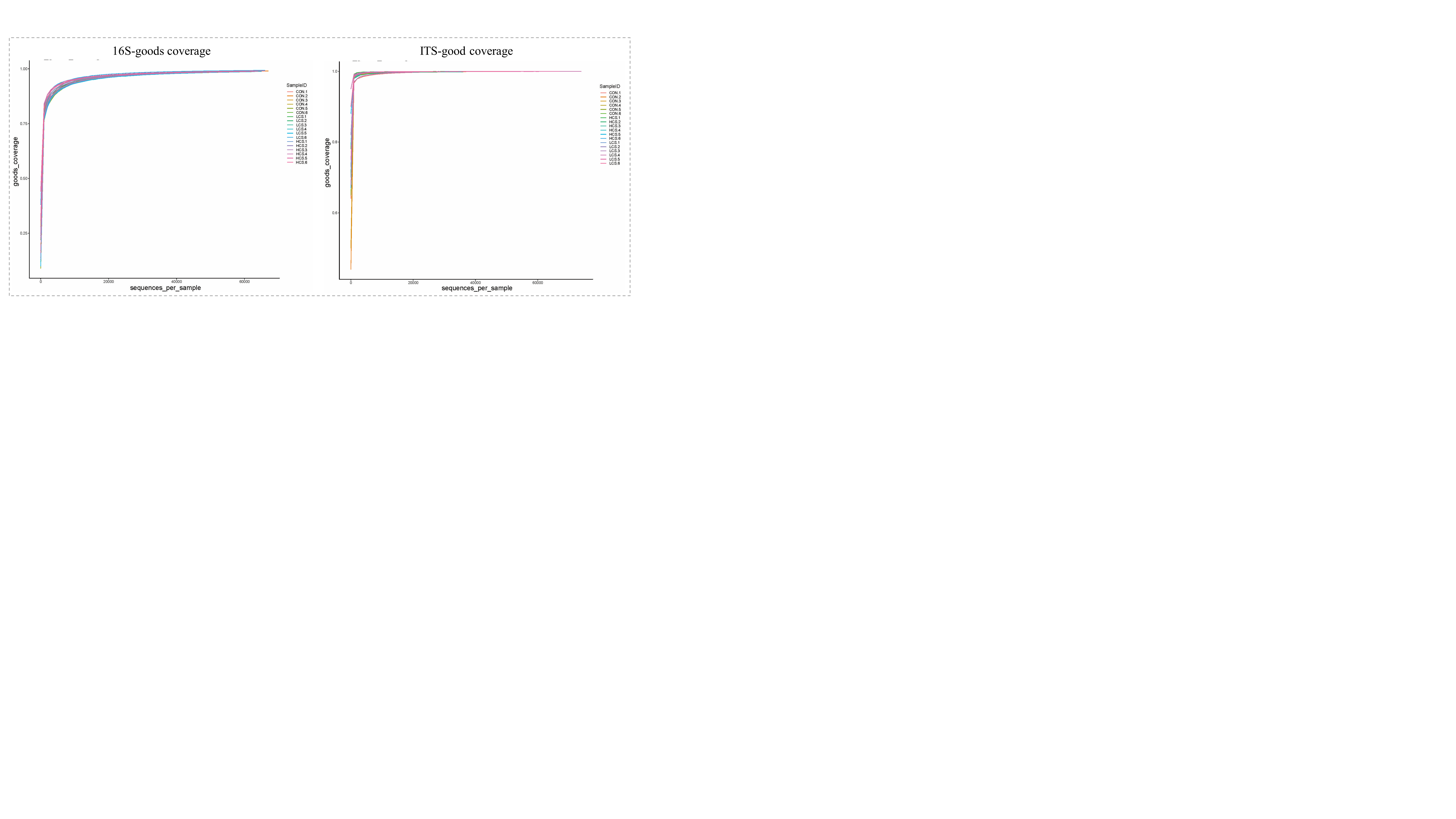
Figure 1. Rarefaction curves of the rumen microbial communities. CON, control group, basal diet; LCS, low CS, basal diet plus 60 mg/kg/d coated CS hydrochloride; HCS, high CS, basal diet plus 120 mg/kg/d coated CS hydrochloride.

Supplementary
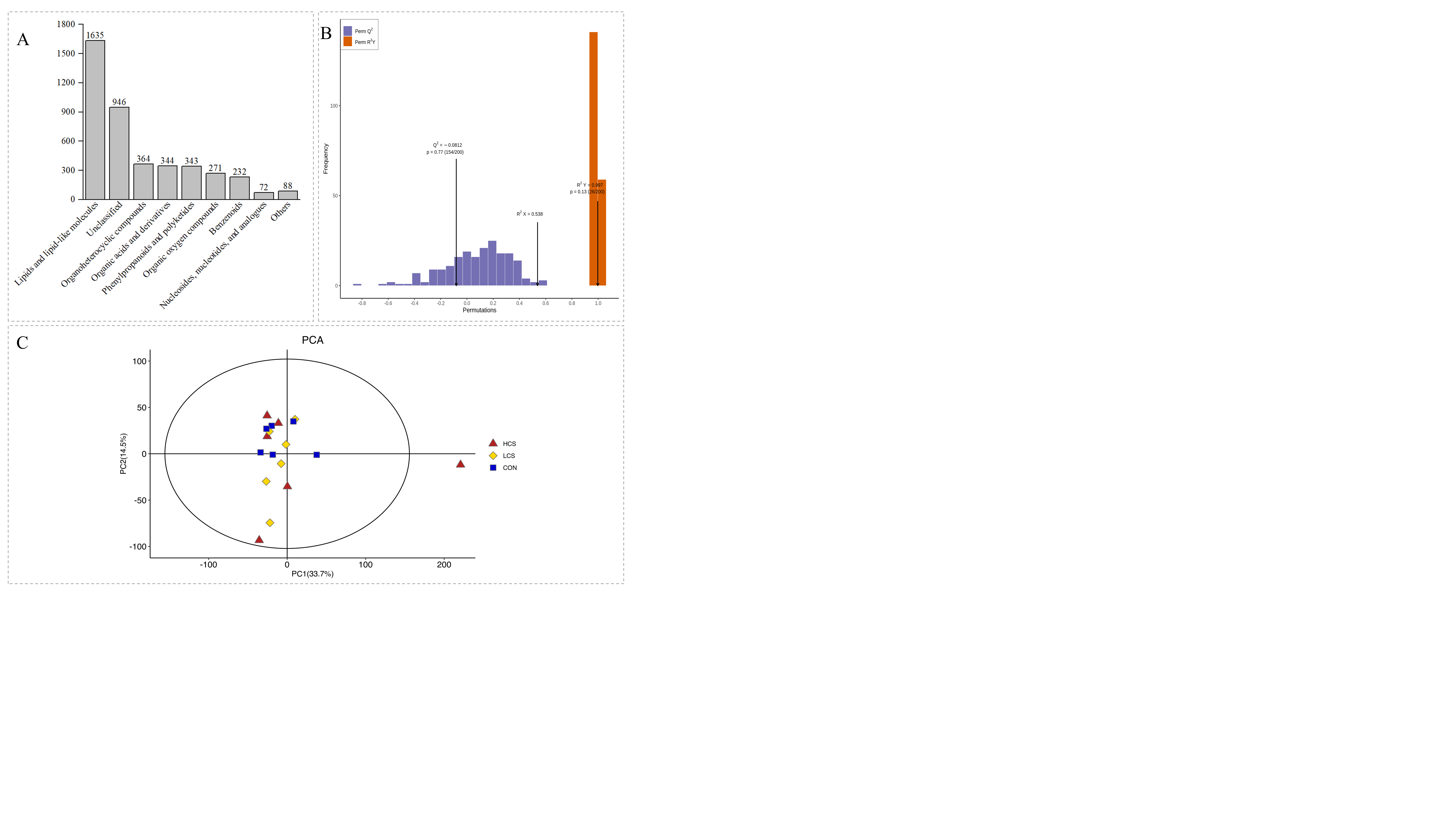
Figure 2. Rumen metabolites of cashmere goats. (A) Numbers of super class of identified metabolites. (B) OPLS-DA permutation test plot. (C)Total PCA plot of the rumen metabolites. CON, control group, basal diet; LCS, low CS, basal diet plus 60 mg/kg/d coated CS hydrochloride; HCS, high CS, basal diet plus 120 mg/kg/d coated CS hydrochloride.
